# Identification of 8 candidate microsatellite instability loci in colorectal cancer and validation of the ACVR2A mechanism in the tumor progression

**DOI:** 10.1038/s41598-024-62753-1

**Published:** 2024-06-19

**Authors:** Jingyu Wang, Zhe Zhang, Hui Liu, Nian Liu, Yucheng Hu, Wenjuan Guo, Xiangzhao Li

**Affiliations:** 1 Molecular Oncology R&D Department, Guangzhou Wondfo Biotechnology Co.,LTD., Guangzhou, China; 2 Department of Pathology, Nanfang Hospital, Southern Medical University, Guangzhou, China

**Keywords:** Colorectal cancer, MSI-H, ACVR2A, PI3K/AKT/mTOR pathway, Biological techniques, Cancer

## Abstract

This study probes the utility of biomarkers for microsatellite instability (MSI) detection and elucidates the molecular dynamics propelling colorectal cancer (CRC) progression. We synthesized a primer panel targeting 725 MSI loci, informed by The Cancer Genome Atlas (TCGA) and ancillary databases, to construct an amplicon library for next-generation sequencing (NGS). K-means clustering facilitated the distillation of 8 prime MSI loci, including activin A receptor type 2A (ACVR2A). Subsequently, we explored ACVR2A’s influence on CRC advancement through in vivo tumor experiments and hematoxylin–eosin (HE) staining. Transwell assays gauged ACVR2A’s role in CRC cell migration and invasion, while colony formation assays appraised cell proliferation. Western blotting illuminated the impact of ACVR2A suppression on CRC’s PI3K/AKT/mTOR pathway protein expressions under hypoxia. Additionally, ACVR2A’s influence on CRC-induced angiogenesis was quantified via angiogenesis assays. K-means clustering of NGS data pinpointed 32 MSI loci specific to tumor and DNA mismatch repair deficiency (dMMR) tissues. ACVR2A emerged as a pivotal biomarker, discerning MSI-H tissues with 90.97% sensitivity. A curated 8-loci set demonstrated 100% sensitivity and specificity for MSI-H detection in CRC. In vitro analyses corroborated ACVR2A’s critical role, revealing its suppression of CRC proliferation, migration, and invasion. Moreover, ACVR2A inhibition under CRC-induced hypoxia markedly escalated MMP3, CyclinA, CyclinD1, and HIF1α protein expressions, alongside angiogenesis, by triggering the PI3K/AKT/mTOR cascade. The 8-loci ensemble stands as the optimal marker for MSI-H identification in CRC. ACVR2A, a central element within this group, deters CRC progression, while its suppression amplifies PI3K/AKT/mTOR signaling and angiogenesis under hypoxic stress.

## Introduction

Colorectal cancer (CRC) ranks as the third most prevalent malignancy and the fourth leading cause of cancer mortality worldwide, with a mortality rate of 1 in 600,000 annually. Current CRC therapeutic protocols—comprising surgical resection, adjuvant chemoradiotherapy, and mesenteric excision—have not substantially extended patient survival^1,2^. However, the advent of targeted therapies has significantly enhanced survival outcomes, thanks to breakthroughs in molecular biology and immunology.

Approximately 15% of non-metastatic and 5% of metastatic CRC cases exhibit microsatellite instability (MSI). The MSI/DNA mismatch repair deficiency (MSI/dMMR) status serves as a prognostic and predictive marker. National Comprehensive Cancer Network (NCCN) guidelines indicate that early-stage CRC patients with high MSI (MSI-H) derive minimal benefit from chemotherapy. Conversely, metastatic CRC patients with MSI/dMMR status respond remarkably well to immune checkpoint inhibitors (ICIs)^3^. Accurate detection of dMMR or MSI tumors is thus pivotal for enhancing cancer screening, diagnosis, and individualized treatment planning^4^. Clinical trials have consistently shown that MSI-H tumor patients respond more favorably to anti-PD-1/PD-L1 therapy^5,6^. The development of targeted therapeutics, including chimeric antibodies, multi-kinase inhibitors, and precision drugs, has enabled the selective inhibition of dysregulated signaling pathways in cancer cells^7,8^. These advancements have curtailed tumor cell proliferation and invasion while minimizing systemic toxicity, offering novel avenues and optimism for efficacious CRC management.

The PI3K/AKT/mTOR signaling cascade is pivotal in modulating the malignant traits of tumors, influencing oncogenesis, metabolic processes, and disease evolution. Diverse human cancers exhibit aberrant activation of this pathway^9,10^. Notably, excessive proliferation of CRC cells can outpace vascular development, precipitating hypoxia, and oxidative stress, which in turn catalyzes a surge in reactive oxygen species (ROS)^11^. ROS serves as a key signal transducer in cellular functions such as proliferation, apoptosis, and angiogenesis. It augments PI3K pathway activity by oxidizing its catalytic subunit (p85), thereby increasing phosphatidylinositol 3,4,5-trisphosphate (PIP3) production and activating downstream effectors like AKT and mTOR^12,13^.

The objective of this investigation is to identify biomarkers for MSI detection in CRC and to dissect the molecular interactions of ACVR2A that influence CRC progression. This endeavor aims to unveil novel insights and therapeutic targets for CRC intervention.

## Methods

### Identification of microsatellite instability (MSI) loci

This research leveraged data from 223 projects within The Cancer Genome Atlas (TCGA) and Wondfo’s proprietary cancer database, which includes Chinese patient data. These projects spanned various tumor types, such as skin, endometrial, gastric, and colorectal cancers (CRC). We utilized genomic data, including whole-exome sequencing and somatic copy number alterations (SCNA), for MSI analysis. Concurrently, we aggregated high MSI (MSI-H) loci cited in the literature for additional scrutiny.

**Figure 1:**
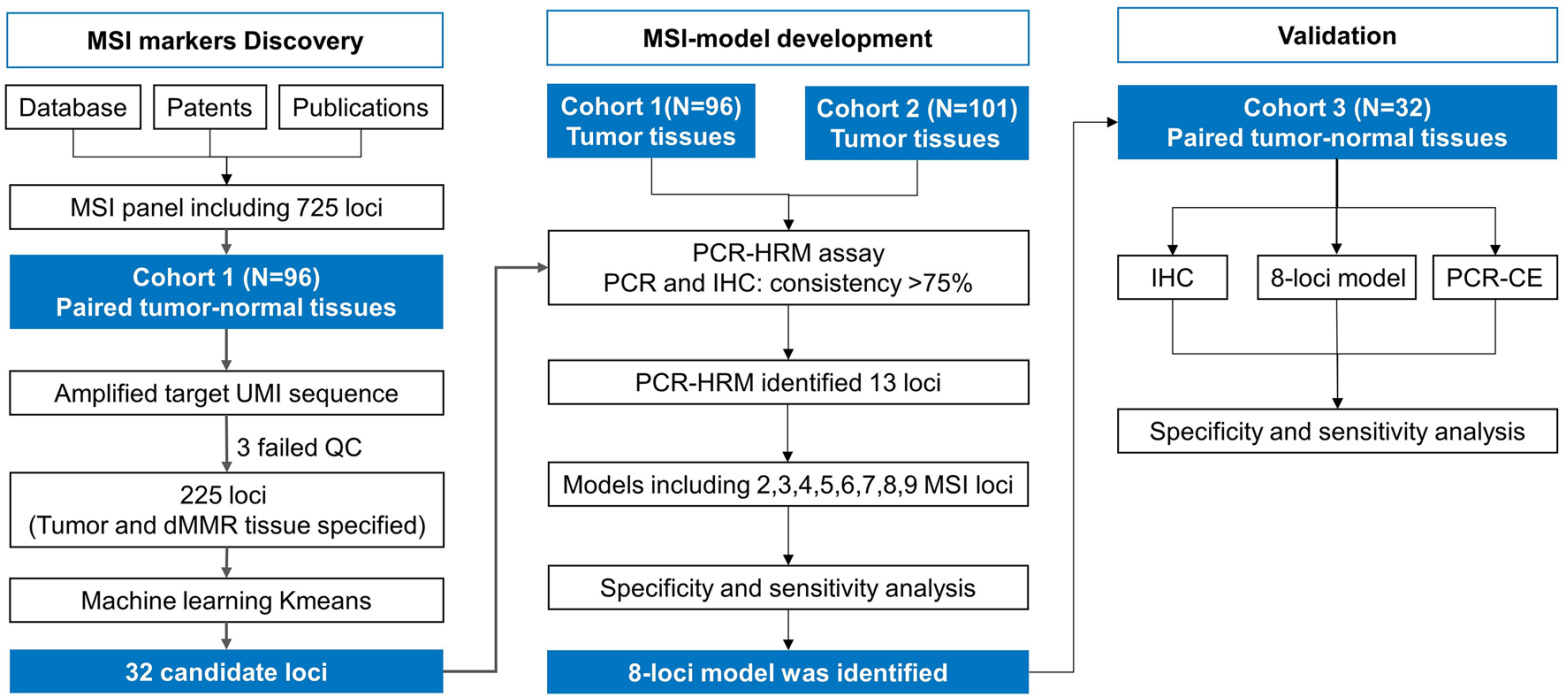
Design of the MSI loci panel.

The study’s methodology is illustrated in Fig. 1. Initially, TCGA and Wondfo’s database were scrutinized to pinpoint MSI-specific loci characterized by genomic repeats ranging from 1 to 5 base pairs (bp), occurring 7 to 13 times. Furthermore, microsatellite loci previously utilized for MSI determination were compiled from diverse public sources, including scholarly articles, patents, and product manuals. This comprehensive approach culminated in the identification of 725 potential MSI loci for subsequent examination. Primers tailored to these loci were also crafted to assemble the unique molecular identifier (UMI)-library.

To pinpoint MSI loci uniquely present in tumor tissues, we collected samples from 96 individuals, comprising both tumor and adjacent non-tumor tissues. These samples were processed into formalin-fixed paraffin-embedded sections and subjected to UMI sequencing (UMI-Seq). Due to DNA extraction failures and quality control issues, three patients’ samples were excluded, leaving 93 matched pairs for analysis.

A meticulous examination of the sequencing data ensued. Initially, we compared the prevalence of MSI loci between tumor and adjacent non-tumor tissues to discern those exclusive to tumors. We then focused on isolating MSI loci characteristic of DNA mismatch repair deficiency (dMMR) tissues. This process yielded 225 MSI loci associated with both tumor presence and dMMR status.

The candidate MSI loci underwent K-means clustering for further refinement. The initial step involved segregating the 225 loci into six categories via hierarchical clustering (refer to Supplementary Fig. 1). Subsequently, K-means clustering was executed. The heatmap analysis directed our attention to loci with pronounced differential abundance between dMMR and proficient MMR (pMMR) tissues. These loci, which exhibited a marked increase in dMMR tissues, are detailed in Supplementary Fig. 2.

### Patient recruitment and sample collection

In this retrospective analysis, 229 colorectal cancer (CRC) patients who underwent radical surgery from January 2018 to December 2021 at our facility were included. Comprehensive mismatch repair (MMR) profiling was conducted for all participants, with subsequent aggregation of the reports. Selection criteria for further investigation stipulated that formalin-fixed paraffin-embedded (FFPE) tumor specimens contain 30–40% tumor cells. From this cohort, tumor-aligned normal paracancerous samples from 96 individuals were earmarked for MSI loci identification. The remaining 133 tumor specimens were allocated to the PCR assay development and validation subgroup (N = 32). Pertinent clinical data encompassing age, gender, and MMR status were meticulously recorded. Sample collection received Southern Medical University Ethics Committee endorsement, adhering to established guidelines and regulations. Informed consent was obtained in writing from all subjects.

### K-means clustering for MSI candidate loci selection

The selection of MSI candidate loci with the strongest tumor association was conducted in stages. Initially, loci that were uniquely present in tumor tissues, as opposed to normal tissues, were identified. This was followed by the isolation of loci that were distinctively present in dMMR tissues when compared to pMMR tissues. K-means clustering was then applied to classify these genes. A subset exhibiting a marked differential signature between dMMR and pMMR tissues was chosen, as indicated by the heatmap generated from these clusters. For the development of the predictive model, 197 samples were designated to the training set, while 32 samples were allocated to the validation set. The validation process entailed comparing the consistency of MSI-H results from the candidate loci with the dMMR status determined by immunohistochemistry (IHC). The model demonstrating the highest sensitivity and specificity was then selected for further validation.

### Cell culture and experimental treatment

Human colorectal cancer (CRC) cell lines HCT116 and HT29, along with the human umbilical vein endothelial cell line ECV-304, were procured from Wuhan Procell Life Science & Technology Co., Ltd. These cells were propagated in Dulbecco’s Modified Eagle Medium (DMEM) enriched with 10% fetal bovine serum (FBS) and 1% penicillin–streptomycin and maintained at 37 °C in a humidified 5% CO_2_ atmosphere. Cells in the logarithmic phase were harvested for experimental assays. HCT116 and HT29 cells underwent transfection with ACVR2A agonist, sh-ACVR2A, and a negative control (NC) vector (GenePharma, Shanghai, China), following the manufacturer’s protocol using Lipofectamine (Invitrogen, Shanghai, China). Post-transfection, cells were categorized into NC, ACVR2A-inhibitor, and ACVR2A-mimic groups for subsequent analyses. Additionally, HCT116 cells were subjected to hypoxic conditions (5% O_2_) and treated with the antioxidant N-acetylcysteine (NAC) and the PI3K inhibitor LY294002 in the NC and ACVR2A inhibitor groups, respectively.

### Subcutaneous tumor xenograft model

Male BALB/c mice, aged 6–8 weeks, were sourced from Henan SCBS Biotechnology Co., Ltd. These mice were allocated randomly into four cohorts, each comprising eight subjects. Subsequently, each mouse was inoculated subcutaneously with 2 × 10^6^ HCT116 cells to establish the xenograft tumor model. Post-inoculation, tumor volumes were quantified at the 10 day mark. The animal study protocols received approval from the Ethics Committee of Southern Medical University (SMULAE-20220309056).

### Hematoxylin–eosin (HE) staining procedure

For histological examination, tumor tissue sections underwent deparaffinization and were immersed in hematoxylin for several minutes. This was succeeded by brief differentiation in acid and ammonia water. Following a thorough rinse in running water for 1 h, the sections were submerged in distilled water, dehydrated in alcohol for 10 min, and stained with eosin for 3 min. After a final alcohol dehydration step and clearance in xylene, the sections were mounted using neutral gum and examined with an optical microscope.

### Transwell migration assays

Transwell assays utilized 24-well plates with polycarbonate membranes (8.0 μm pore size). HCT116 and HT29 cells were seeded in the upper chamber in 100 μL of serum-free medium at a density of 1 × 10^5^ cells/well. The lower chamber received 600 μL of the complete medium as a chemoattractant. Following a 37 h incubation at 37 °C, non-migratory cells were cleared from the membrane surface with cotton swabs. Migratory cells were then fixed with 4% paraformaldehyde, stained with 0.1% crystal violet, and imaged using an inverted fluorescence microscope.

### Colony formation assays

For colony formation assays, HCT116 and HT29 cells in logarithmic growth were trypsinized, counted, and plated in humidified incubators at 37 °C. After approximately two weeks, once visible colonies had developed, the medium was removed, and cells were washed with PBS, fixed in methanol for 15 min, air-dried, and stained with crystal violet for 30 min. Post-staining, colonies were imaged and quantified.

### Western blot analysis

Protein extraction from HCT116 cells was achieved using RIPA lysis buffer, supplemented with protease and phosphatase inhibitors. The Pierce™ BCA Protein Assay Kit facilitated protein concentration quantification, adhering to the manufacturer’s guidelines. Electrophoresis involved a 10% separating gel and a 5% stacking gel within a chamber containing 1× running buffer. Post-electrophoresis, proteins were transferred to a PVDF membrane. Blocking occurred with 5% skim milk at room temperature for 1 h, followed by overnight incubation at 4 °C with primary antibodies targeting NOX4, p-PI3K, p-mTOR, MMP3, CyclinA, CyclinD1, HIF1α, and GAPDH. Secondary antibodies were applied at room temperature for 90 min. Chemiluminescence imaging concluded the process.

### Angiogenesis assay

ECV-304 cells, co-cultured with HCT116 cells, underwent serum starvation in basal medium for 8 h upon reaching over 80% confluence. Matrigel, thawed at 4 °C, was applied to a pre-chilled 24-well plate and allowed to solidify at 37 °C for 30 min. ECV-304 cells, trypsinized and suspended in serum-free medium (5 × 10^5^ cells/100 μL), were added to the gelled Matrigel. Sprouting was monitored and captured under an inverted microscope within 4 h.

### Statistical analysis

Data analysis and visualization were conducted using GraphPad Prism 9.0. Normally distributed data were presented as mean ± standard error of the mean (SEM). Comparative analysis employed independent-samples t-tests for two groups and one-way ANOVA for multiple groups. A p-value of less than 0.05 denoted statistical significance.

## Results

### Optimal loci combination identification

Thirteen genes, including ACVR2A, DIDO1, MRE11, KMT2C-MLL3, TGFBR2, RPL22, CENPQ, CADM3, PSIP1, PIM1, LRIG2, SLC22A9, and ZNF2, were scrutinized for their potential as MSI markers. As delineated in Supplementary Table 1, all genes exhibited a specificity of 100%, with sensitivities ranging from 70.83 to 90.97%. To construct an MSI detection model with superior sensitivity and specificity, combinations of multiple loci were evaluated. A tissue was deemed MSI-High (MSI-H) upon detecting instability in at least two candidate mononucleotide repeat loci. Supplementary Table 2 presents the optimal combinations, showcasing varying consistencies, sensitivities, and specificities. The 2-loci combination, while maintaining 100% specificity, offered the lowest sensitivity at 81.94%. In contrast, the 8-loci combination—comprising ACVR2A, TGFBR2, SLC22A9, DIDO1, LRIG2, MRE11, CENPQ, and PSIP1—achieved the highest sensitivity at 96.53%.

The efficacy of the 8-loci tumor MSI model was validated using tumor samples from 32 patients without corresponding normal tissue. The validation results confirmed that, relative to the dMMR or pMMR status ascertained by pathologists, the 8-loci MSI model identified dMMR tumors with 100% specificity, sensitivity, positive predictive value, and negative predictive value (Table 1).

**Table 1:** Sensitivity and specificity of the 8-loci model compared with IHC results.

		8 Loci-model				Promega			
		MSI-H	MSI-L/MSS	Sensitivity	Specificity	MSI-H	MSI-L/MSS	Sensitivity	Specificity
IHC	dMMR	16	0	100.00%	100.00%	16	0	100.00%	93.75%
	pMMR	0	16			1	15		

### ACVR2A’s role in CRC development

**Figure 2:**
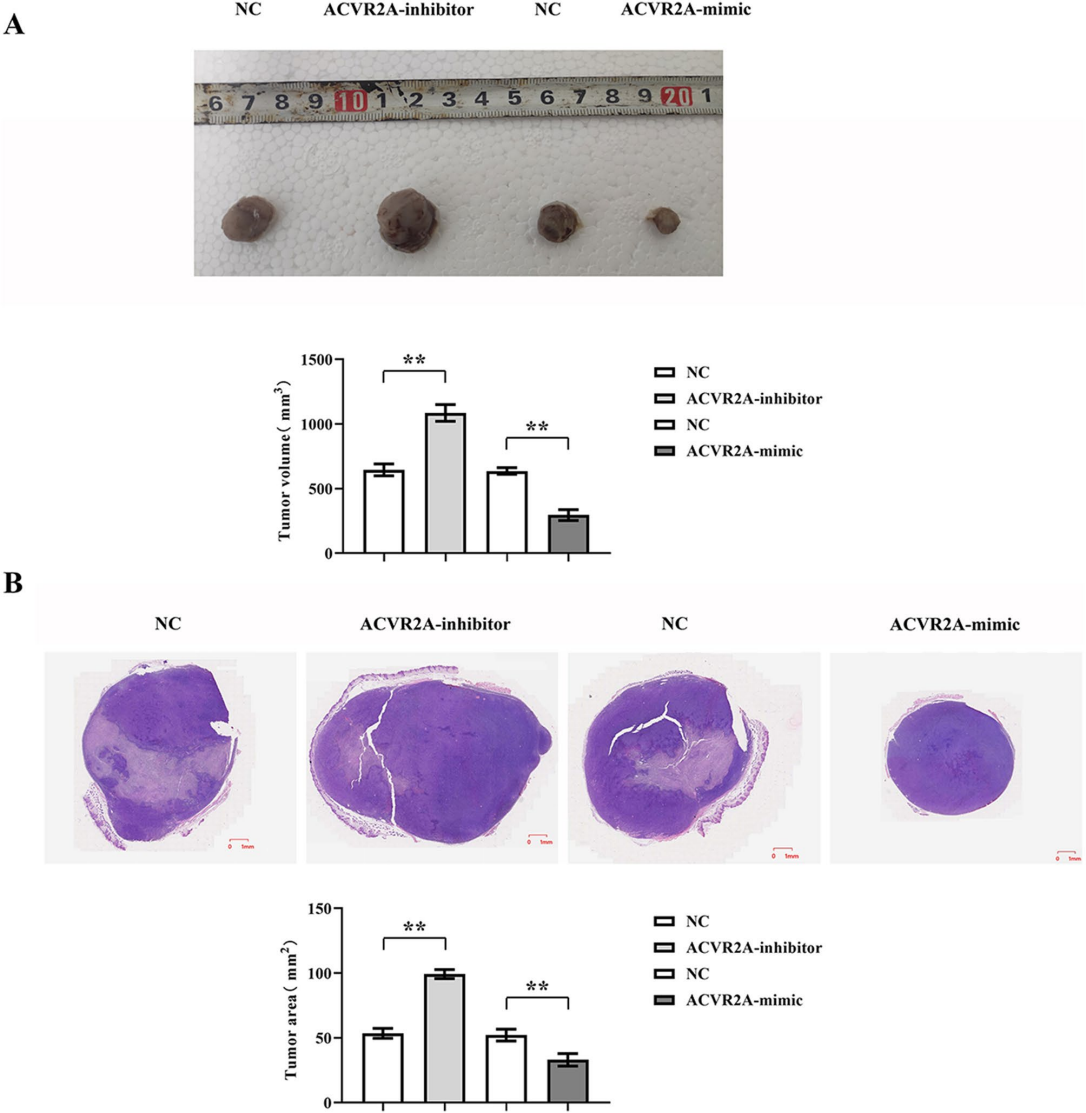
ACVR2A inhibits CRC development. (**A**) Subcutaneous tumor xenograft experiment results and tumor volume statistics in nude mice; (**B**) Hematoxylin and eosin (HE) staining results and tumor area statistics.

The subcutaneous tumor xenograft experiment revealed that, relative to the negative control (NC) group, tumor volume markedly escalated in the ACVR2A-inhibitor group but substantially diminished in the ACVR2A-mimic group (Fig. 2A). Hematoxylin–eosin (HE) staining demonstrated a significant increase in tumor area in the ACVR2A-inhibitor group and a decrease in the ACVR2A-mimic group compared to the NC group (Fig. 2B). These findings suggest that ACVR2A may play a role in impeding CRC progression.

### ACVR2A’s influence on CRC cell migration and invasion

**Figure 3:**
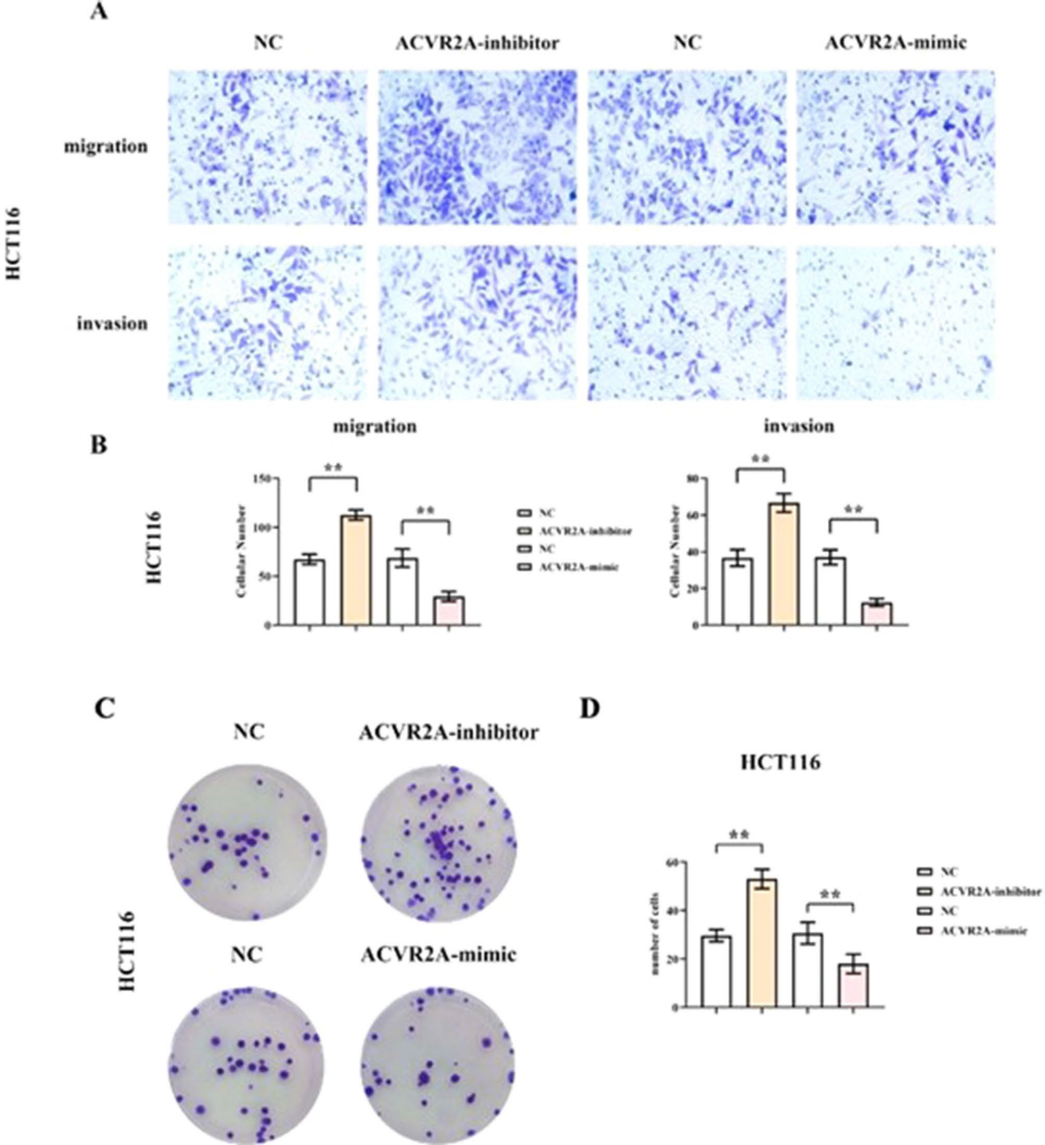
ACVR2A suppresses migration, invasion, and proliferation of CRC cells (HCT116). (**A**) Transwell assay results of HCT116 cells; (**B**) Quantification of migrating and invading HCT116 cells; (**C**) Colony formation assay results of HCT116 cells; (**D**) Quantification of HCT116 cell colonies.

Transwell assays were employed to assess ACVR2A’s impact on CRC cell migration and invasion. The results indicated a significant induction of cell migration and invasion in the ACVR2A-inhibitor group, while a notable reduction was observed in the ACVR2A-mimic group, in comparison to the NC group (Fig. 3A,B, Supplementary Fig. 3A,B). This implies that ACVR2A has the potential to inhibit the migratory and invasive capabilities of CRC cells.

### Impact of ACVR2A on CRC cell proliferation

Colony formation assays indicated a significant increase in colony numbers in the ACVR2A-inhibitor group compared to the negative control (NC) group, while a notable decrease was observed in the ACVR2A-mimic group (Fig. 3C,D, Supplementary Fig. 3C,D). This suggests that ACVR2A plays a role in restraining the proliferation of CRC cells.

### ACVR2A’s effect on PI3K/AKT/mTOR pathway under hypoxia

**Figure 4:**
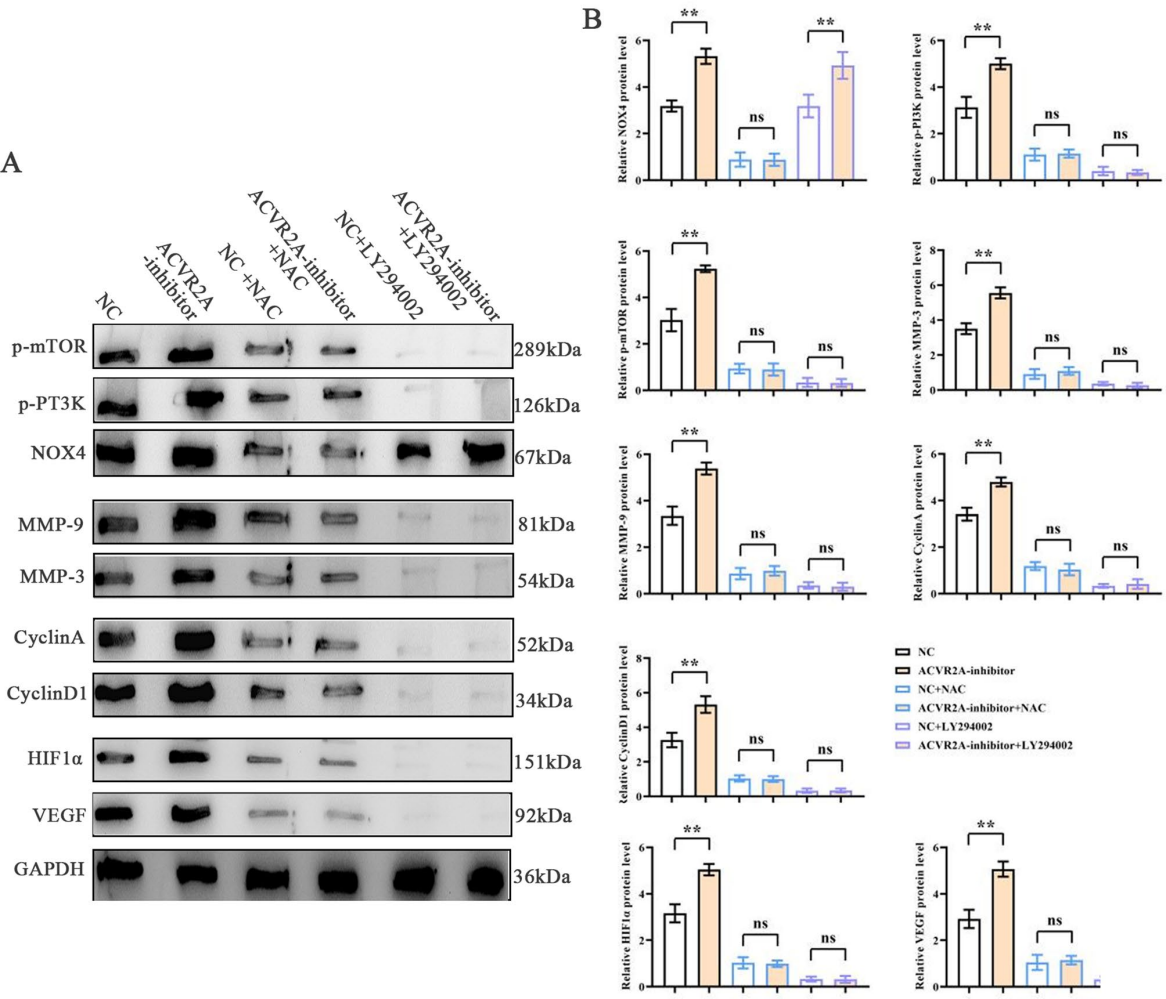
ACVR2A suppression enhances the expression of PI3K/AKT/mTOR signaling pathway-related proteins under hypoxic conditions. (**A**) Western blot analysis of NOX4, phospho-PI3K (p-PI3K), phospho-mTOR (p-mTOR), MMP3, MMP9, Cyclin A, Cyclin D1, HIF1α, and VEGF protein levels; (**B**) Quantification of relative protein expression levels of NOX4, p-PI3K, p-mTOR, MMP3, MMP9, Cyclin A, Cyclin D1, HIF1α, and VEGF.

The proliferation of CRC cells can lead to hypoxic conditions in vivo. Under such conditions, HCT116 cells were treated with N-acetylcysteine (NAC) and the PI3K inhibitor LY294002. Western blot analysis showed that the inhibition of ACVR2A significantly upregulated the expression of proteins associated with the PI3K/AKT/mTOR signaling pathway, including NOX4, p-PI3K, p-mTOR, MMP3, CyclinA, CyclinD1, and HIF1α, compared to the NC group. The addition of NAC led to a significant reduction in these proteins in both the NC and ACVR2A-inhibitor groups. Similarly, LY294002 treatment resulted in decreased expression of p-PI3K, p-mTOR, MMP3, CyclinA, CyclinD1, and HIF1α in both groups, while NOX4 levels remained unchanged (Fig. 4).

### ACVR2A inhibition and PI3K/AKT/mTOR pathway-induced angiogenesis

**Figure 5:**
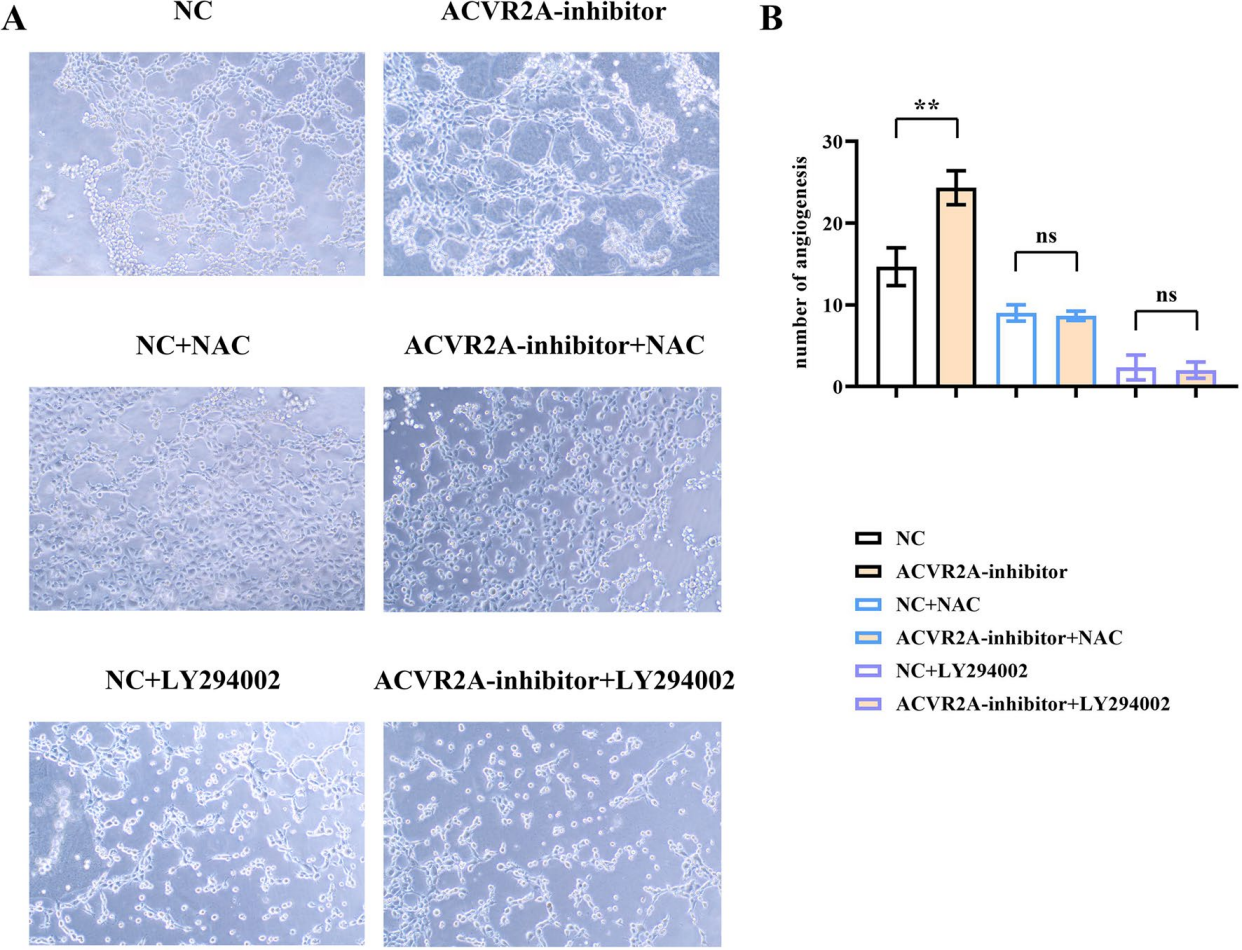
ACVR2A suppression activates the PI3K/AKT/mTOR signaling pathway and promotes angiogenesis under hypoxic conditions. (**A**) Angiogenesis assay results; (**B**) quantification of newly formed blood vessels.

The angiogenesis assay revealed a significant increase in neovascularization in the ACVR2A-inhibitor group compared to the negative control (NC) group. Notably, the administration of N-acetylcysteine (NAC) or the PI3K inhibitor LY294002 markedly reduced the formation of new blood vessels in both the NC and ACVR2A-inhibitor groups (Fig. 5). These findings imply that the suppression of ACVR2A under hypoxic conditions encountered in CRC may trigger angiogenesis through the PI3K/AKT/mTOR signaling pathway.

## Discussion

CRC ranks among the most prevalent malignancies of the digestive tract globally, with a mortality rate on the rise. Accurate assessment of MSI status is crucial for tailoring appropriate therapeutic strategies for CRC patients. The genomic landscape, replete with myriad microsatellite loci, exerts a profound influence on treatment efficacy. Our investigation led to the development of a PCR-amplifiable biomarker suite, encompassing eight microsatellite loci, designed to precisely and reliably ascertain the MSI status in CRC tumor specimens. This biomarker suite demonstrated a specificity of 100% across both training and validation cohorts, coupled with sensitivities of 96.53% and 100%, respectively.

In the course of this research, an initial pool of 725 MSI loci was curated from existing databases and pertinent literature. A comparative analysis of 93 matched tumor and adjacent non-tumor tissue pairs was conducted to pinpoint tumor-specific loci. To mitigate amplification bias and bolster sequencing fidelity, UMI-Seq was employed, significantly diminishing background interference while preserving a high positive predictive value and sensitivity. The ensuing data underwent K-means clustering analysis, which identified 32 loci exhibiting marked differential representation between dMMR and pMMR tissues.

An octet of gene loci emerged as the most efficacious in discerning MSI-H tissues, exhibiting a sensitivity of 96.53% and a specificity of 100% within the training dataset, and maintaining 100% sensitivity and specificity in the validation cohort. These findings underscore the potential utility of this loci combination for MSI-H detection in colorectal cancer patients. Notably, the panel’s minimal sample requirement—merely 4–6 FFPE sections—enhances its practicality for clinical application. The data suggest that this panel could supplant existing MSI-H identification methods in clinical settings, although further validation with a more extensive sample set is warranted.

**Figure 6:**
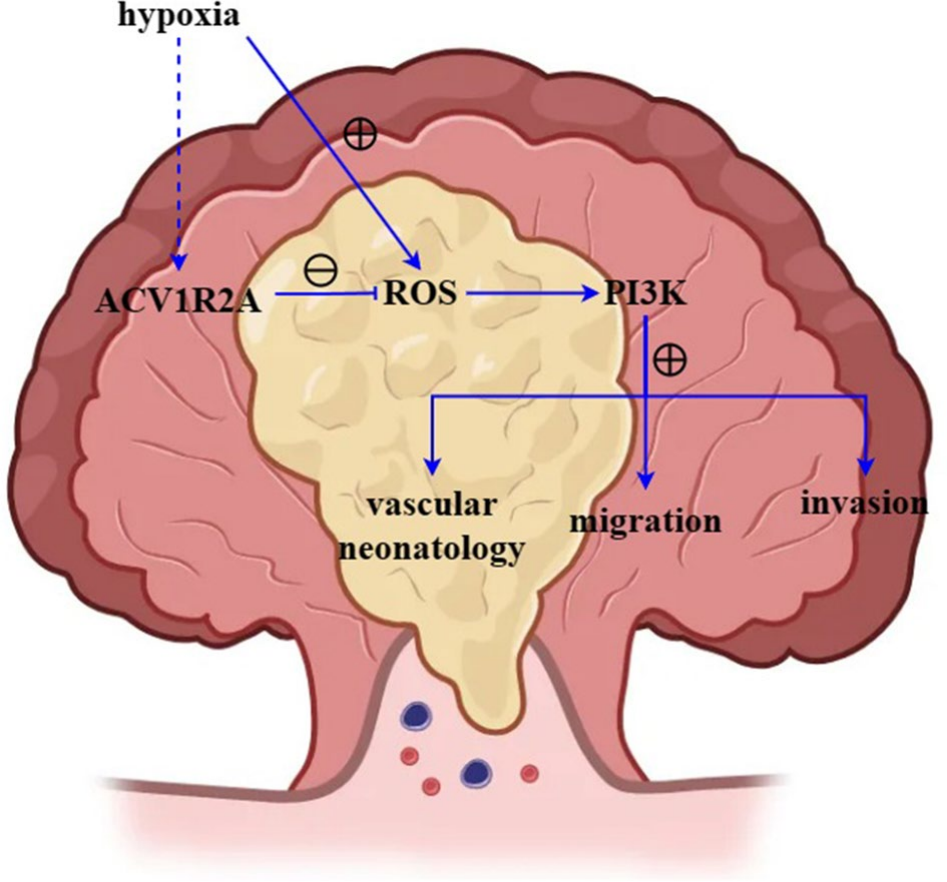
ACVR2A suppression activates the PI3K/AKT/mTOR signaling pathway in the hypoxic CRC microenvironment, accelerating CRC progression.

Within the panel, the influence of TGFBR2^14^, SLC22A9^15^, DIDO1^16^, and MRE11^17^ on CRC cell microsatellite instability has been documented. Among the previously unreported loci, ACVR2A demonstrated pronounced sensitivity. Our study contributes novel insights into the clinicopathological role of ACVR2A in CRC progression. ACVR2A, a member of the TGF-β receptor family, orchestrates a myriad of biological functions, including embryogenesis, cellular proliferation, differentiation, tissue repair, and immune modulation. Research indicates that ACVR2A-mediated signaling may modulate ROS production and elimination through redox equilibrium regulation. Furthermore, ACVR2A influences cellular redox homeostasis and oxidative stress pathways, notably the Nrf2 signaling cascade, impacting ROS levels (Fig. 6)^18–20^. Crucially, ACVR2A may act as a tumor suppressor in CRC, impeding tumorigenesis and differentiation. Loss of ACVR2A has been implicated in the advancement and metastasis of colon cancer. Our investigations, encompassing subcutaneous tumor xenografts and HE staining analyses, revealed that, relative to the NC group, tumor volume and area markedly escalated in the ACVR2A-inhibitor cohort, whereas a significant diminution was observed in the ACVR2A-mimic group, suggesting ACVR2A’s inhibitory effect on CRC progression. Further mechanistic exploration revealed that, compared to the NC group, the ACVR2A-inhibitor cohort exhibited a substantial increase in migratory and invasive cell populations, as well as colony formation, thereby affirming ACVR2A’s role in thwarting CRC evolution by curtailing cell proliferation, migration, and invasion.

Hypoxia in CRC has been shown to induce oxidative stress and ROS production, which play a pivotal role in the delicate balance between tumor cell survival and death, as well as in various aspects of tumor development, including cell proliferation, metastasis, cell cycle progression, and senescence^21–24^. Notably, ROS activates the PI3K/AKT/mTOR pathway, which contributes to the excessive proliferation of cancer cells^25,26^. AKT, also known as protein kinase B (PKB), is a serine/threonine kinase downstream of PI3K that regulates cellular responses to external stimuli and controls cell proliferation and survival through various intracellular signaling cascades^27–29^. Furthermore, mTOR, a serine/threonine kinase belonging to the PI3K-related kinase family, plays a crucial role in cell growth, apoptosis, autophagy, and metabolism. In this study, we investigated whether ACVR2A acts through the ROS/PI3K pathway. To mimic the in vivo CRC environment, HCT116 cells were cultured under hypoxic conditions and treated with or without *N*-acetylcysteine (NAC) and LY294002. As expected, western blot analysis revealed that under hypoxic conditions, ACVR2A suppression in CRC markedly induced the expression of MMP3, Cyclin A, Cyclin D1, and HIF1α, accompanied by enhanced PI3K/AKT/mTOR signaling in CRC cells.

Angiogenesis is a fundamental hallmark of cancer progression and plays a critical role in CRC progression and metastasis^30^. Understanding the signaling pathways that regulate angiogenesis in CRC is crucial for identifying potential therapeutic targets^31^. To date, no studies have investigated the impact of ACVR2A expression on angiogenesis in colorectal cancer. In this study, we evaluated the effect of ACVR2A on angiogenesis associated with colorectal cancer cells. Interestingly, ACVR2A suppression under hypoxic conditions markedly enhanced angiogenesis in the presence of CRC. Both the antioxidant NAC and the PI3K inhibitor LY294002 dramatically inhibited angiogenesis, suggesting that ROS/PI3K/AKT/mTOR signaling plays a key role in ACVR2A suppression-induced angiogenesis. Notably, LY294002 exhibited a stronger inhibitory effect than NAC. One possible explanation is that LY294002, as a potent PI3K inhibitor, also blocked the function of glial cell line-derived neurotrophic factor (GDNF) [DY1], which stimulates VEGF-independent angiogenesis.

In conclusion, the 8-loci model constructed in this study demonstrated 100% sensitivity and specificity in identifying MSI-H tissues in colorectal cancer. ACVR2A inhibits CRC proliferation, migration, and invasion, and its suppression under hypoxic conditions stimulates the PI3K/AKT/mTOR signaling pathway and angiogenesis. The activation of the PI3K/AKT/mTOR signaling pathway in CRC by ACVR2A suppression under hypoxic conditions further induces angiogenesis, thereby promoting the progression of CRC.

### Supplementary Information

Supplementary Figure 1.

Supplementary Table 1.

Supplementary Table 2.

Supplementary Information.

## Data Availability

Sequence data that support the findings of this study have been deposited in the GEO with the primary accession code GSE246793.
