# Supplementary material for: Identification of 8 candidate microsatellite instability loci in colorectal cancer and validation of the ACVR2A mechanism in the tumor progression
Source: Sci Rep. 2024 Jun 19;14:14145. doi: 10.1038/s41598-024-62753-1 (PMC11187151; doi:10.1038/s41598-024-62753-1)
Supplement: Supplementary file 1 — Supplementary Figure 1. [file 41598_2024_62753_MOESM1_ESM.docx]

Supplement Figures


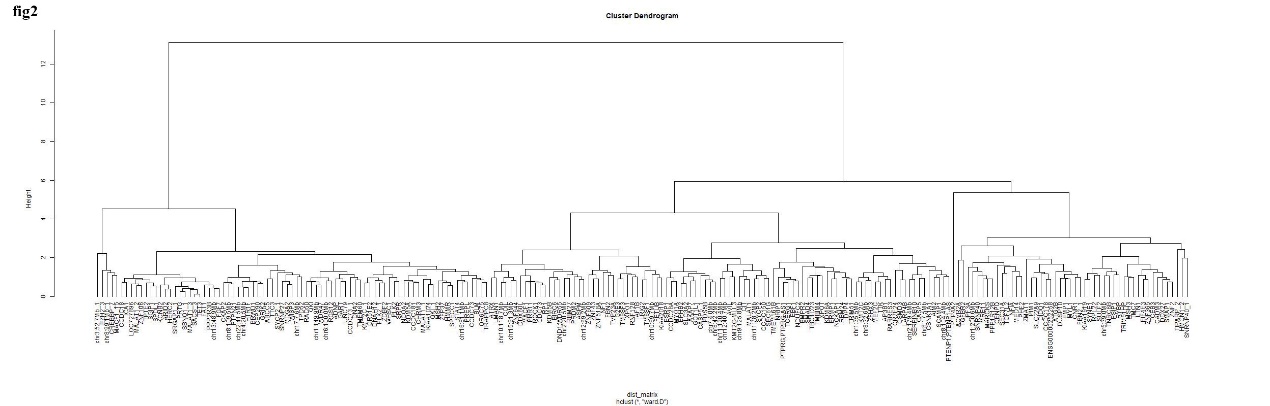


Supplement Figure 1: Hierarchical clustering of 225 loci (6 groups)


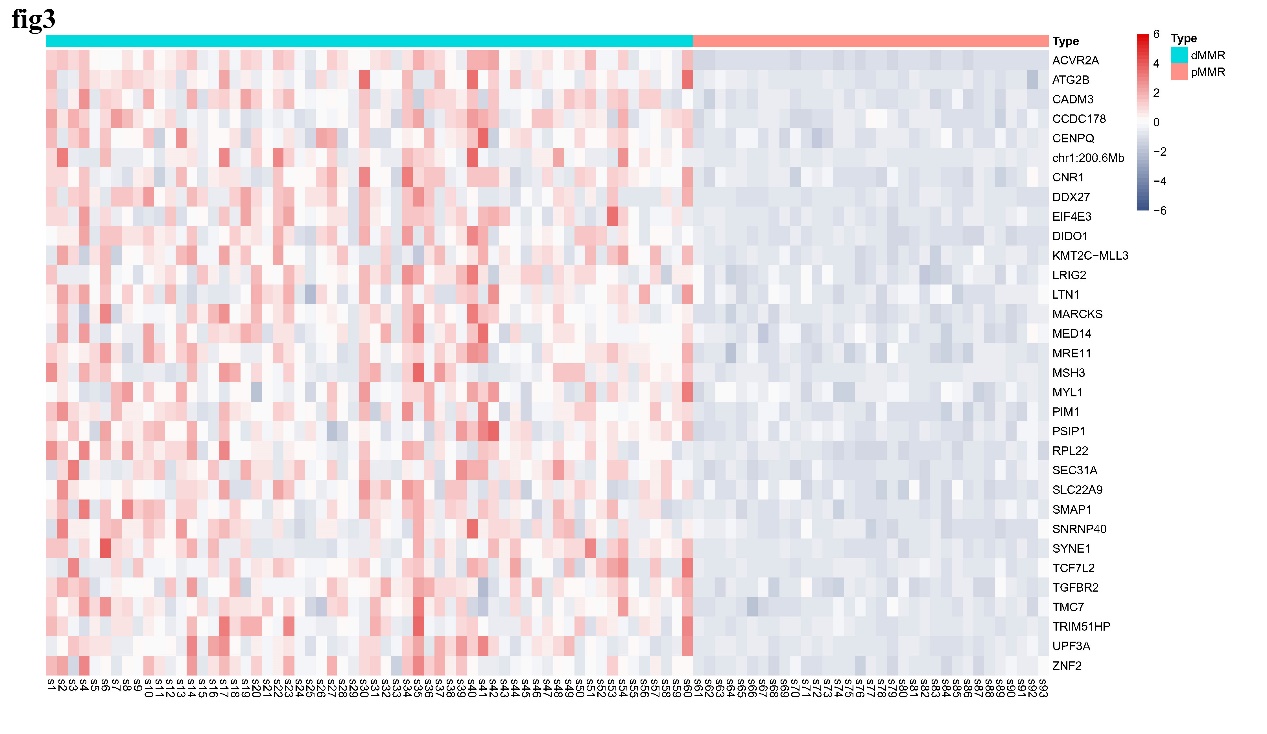


Supplement Figure 2: Loci with the greatest difference in abundance between dMMR and pMMR tissues


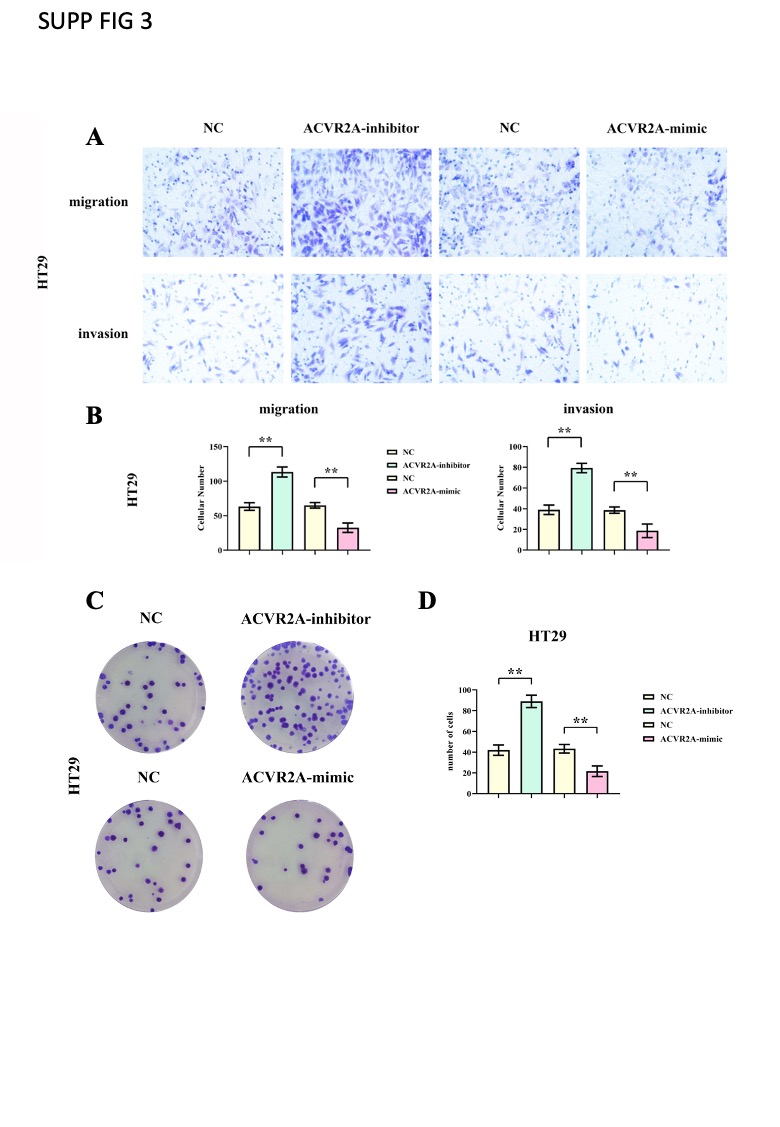


Supplement Figure 3. ACVR2A could suppress the migration, invasion, and proliferation of CRC cells (HT29).

A) Results of Transwell assay of HT29 cells; B) Statistics of the number of migrating and invading HT29 cells; C) Results of colony formation assay of HT29 cells; D) Statistics of the number of colonies of HT29 cells.
