# Supplementary material for: Identification of 8 candidate microsatellite instability loci in colorectal cancer and validation of the ACVR2A mechanism in the tumor progression
Source: Sci Rep. 2024 Jun 19;14:14145. doi: 10.1038/s41598-024-62753-1 (PMC11187151; doi:10.1038/s41598-024-62753-1)

Fig.7A_p-mTOR_shine_raw

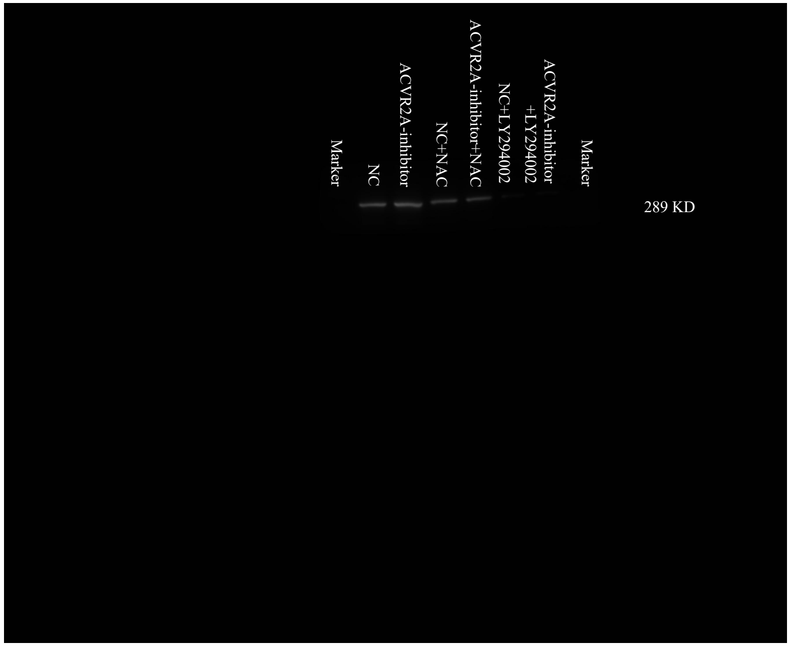

Fig.7A_p-mTOR_bright

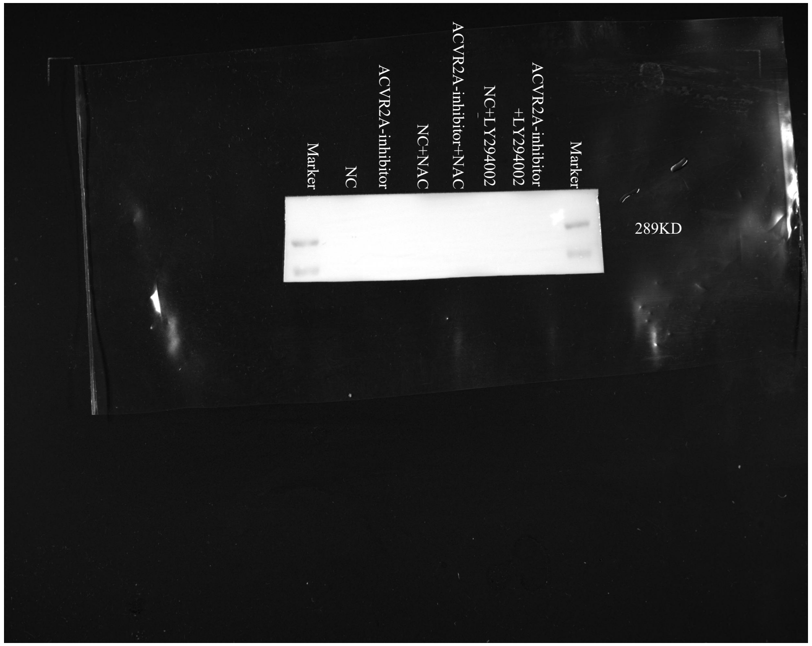

Fig.7A_p-mTOR_shine

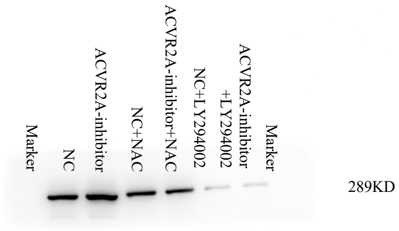

Fig.7A_p-mTOR_merge

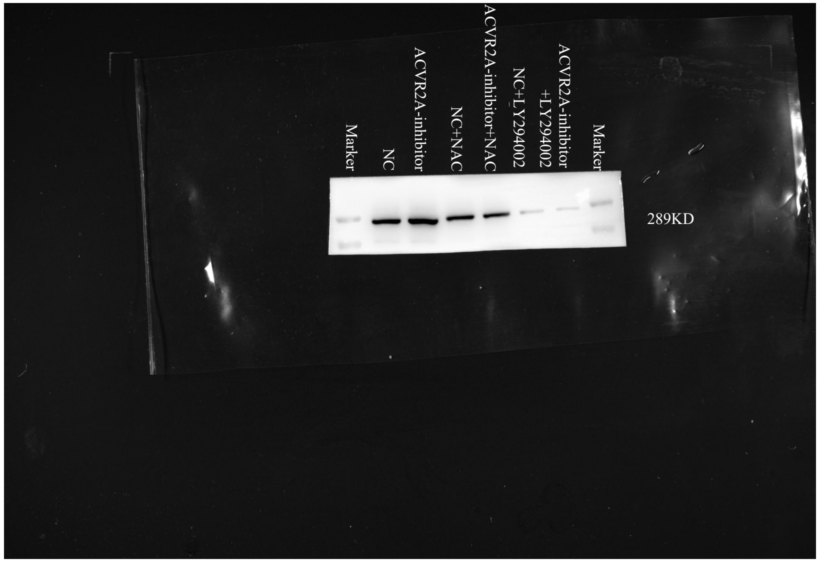

Fig.7A_p-PT3K_shine_raw

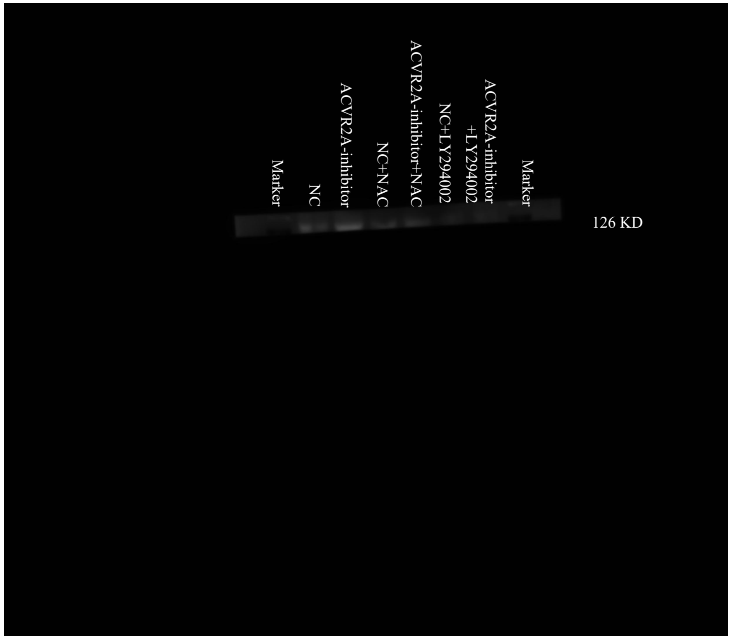

Fig.7A_p-PT3K_bright

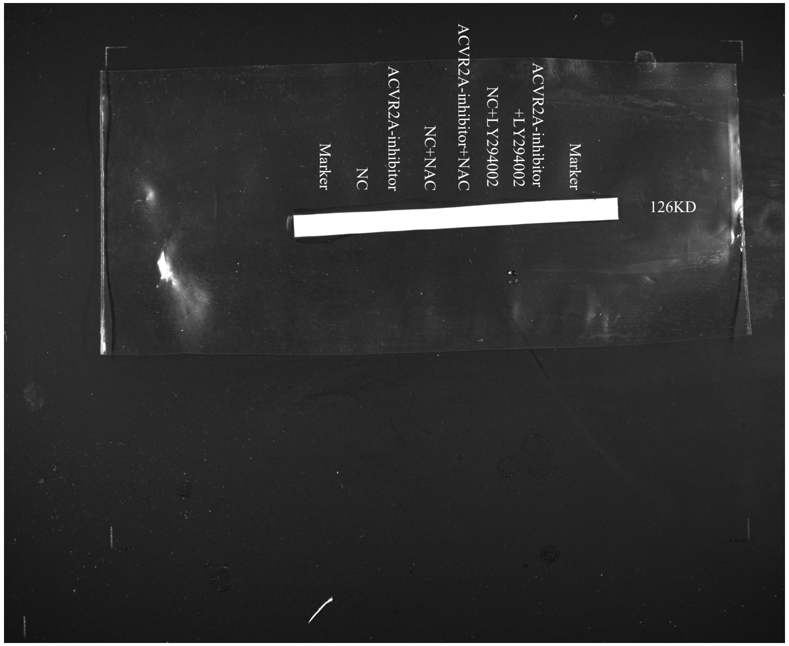

Fig.7A_p-PT3K_shine

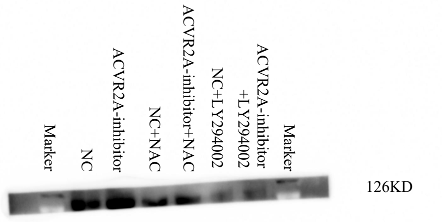

Fig.7A_p-PT3K_merge

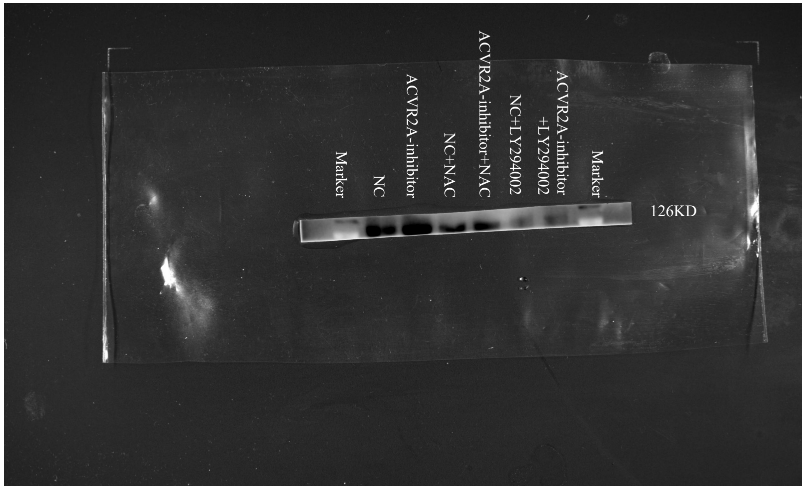

Fig.7A_NOX4_shine_raw

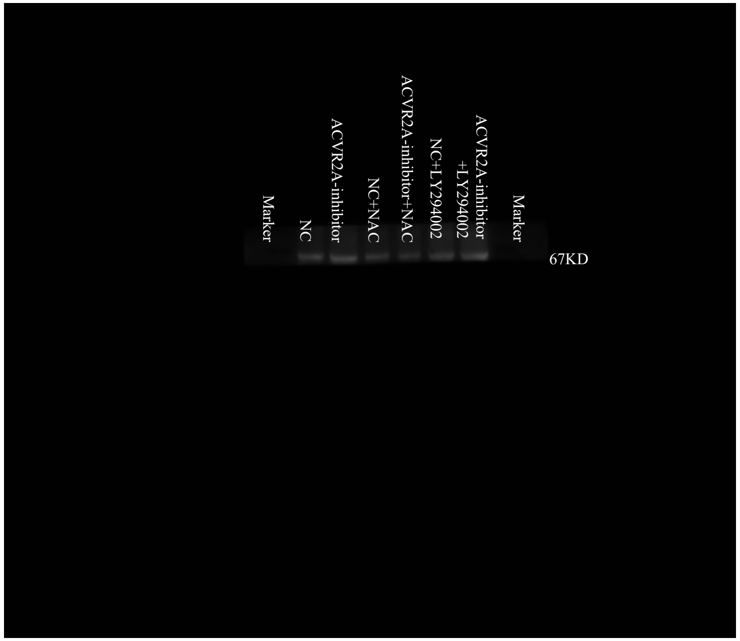

Fig.7A_NOX4_bright

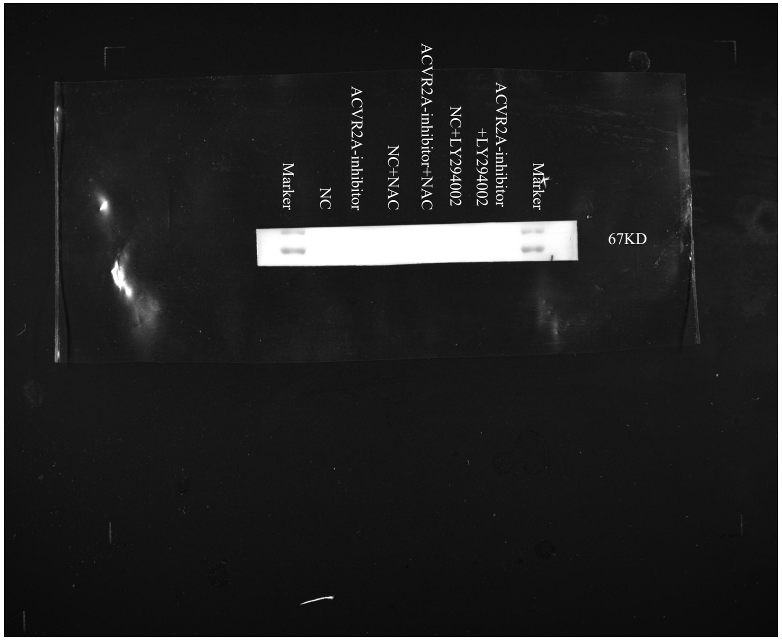

Fig.7A_NOX4_shine

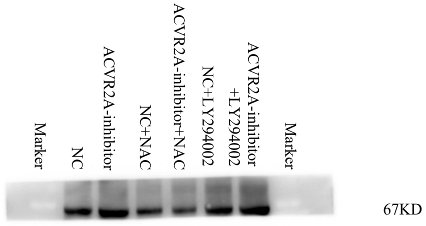

Fig.7A_NOX4_merge

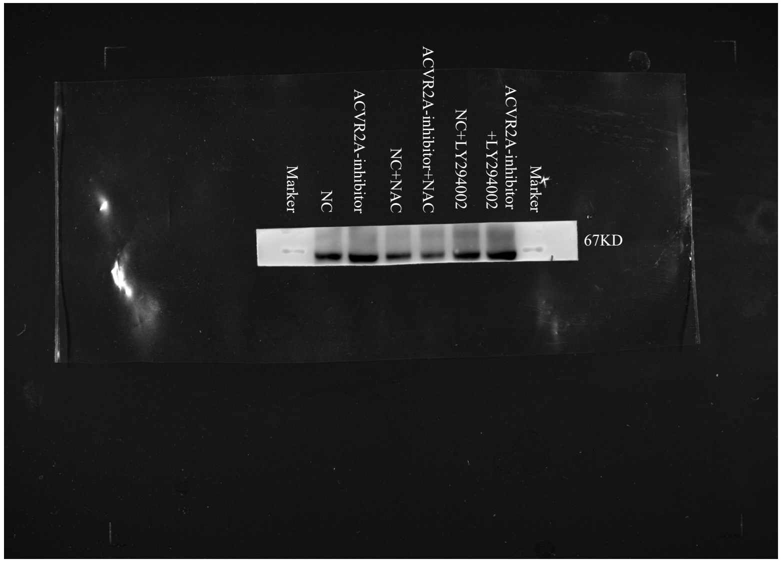

Fig.7A_MMP-3_shine_raw

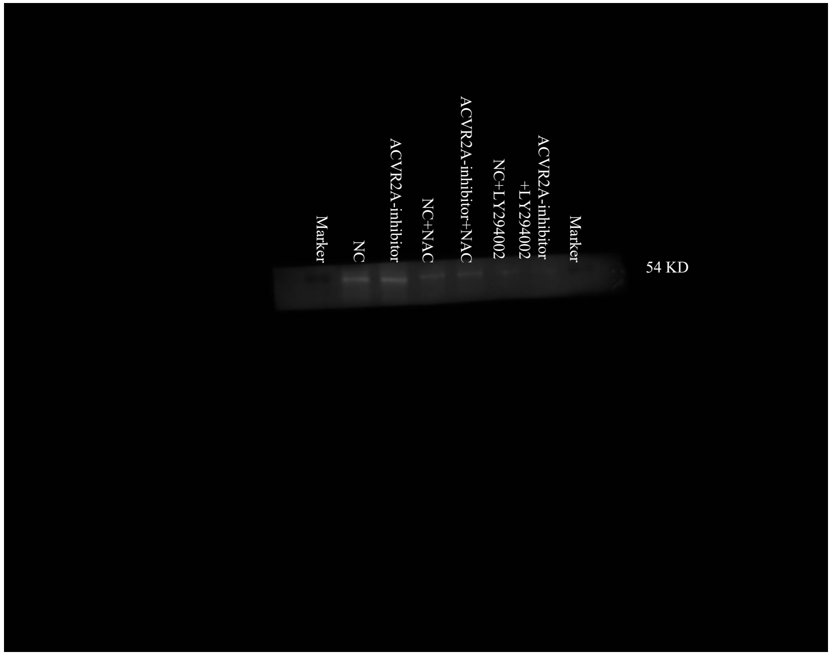

Fig.7A_MMP-3_bright

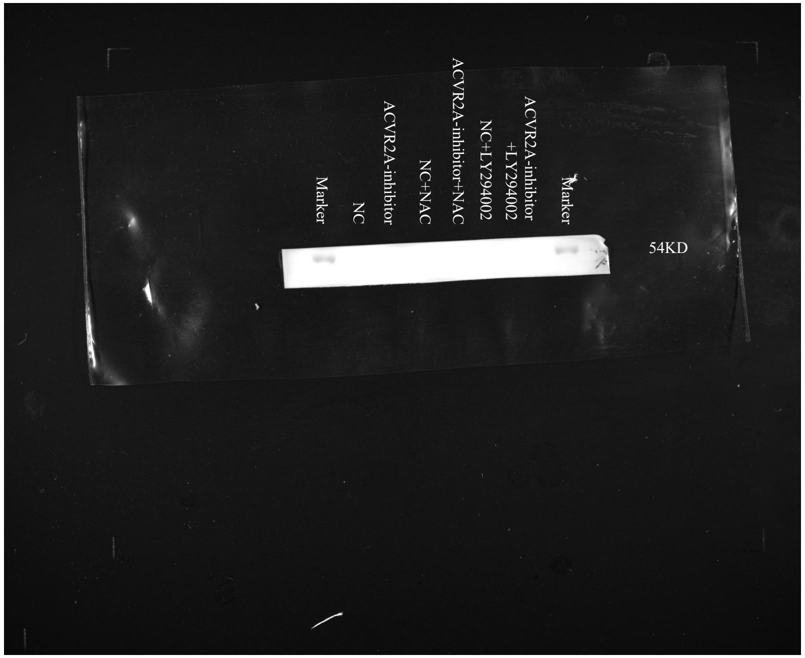

Fig.7A_MMP-3_shine

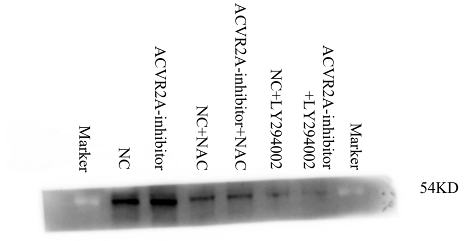

Fig.7A_MMP-3_merge

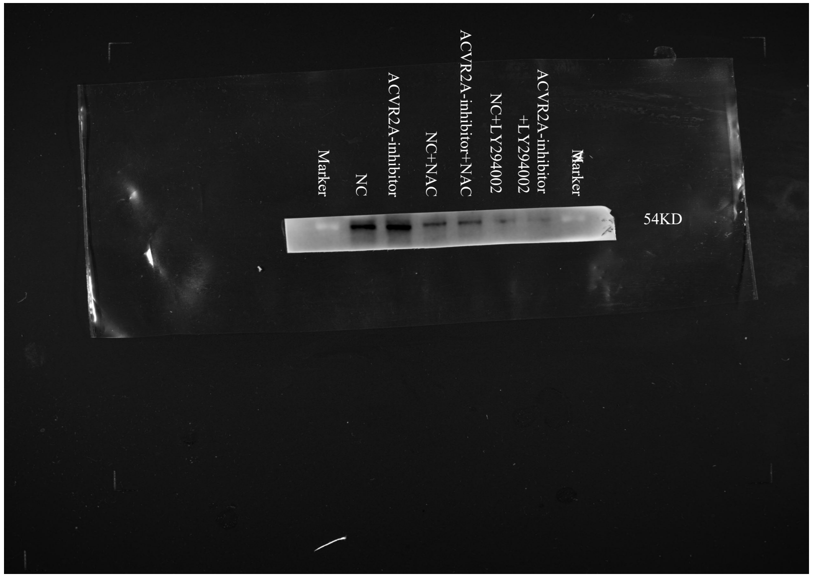

Fig.7A_CyclinA_shine_raw

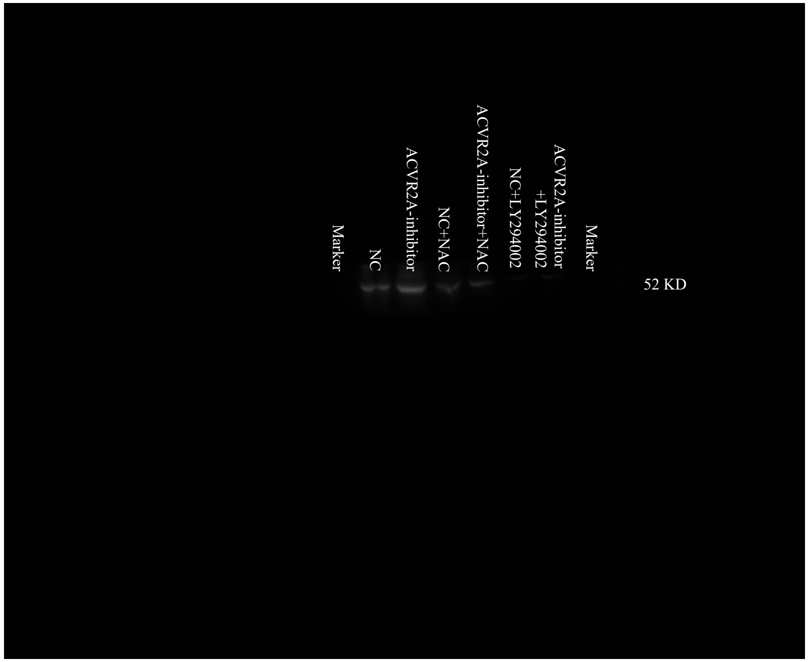

Fig.7A_CyclinA_bright

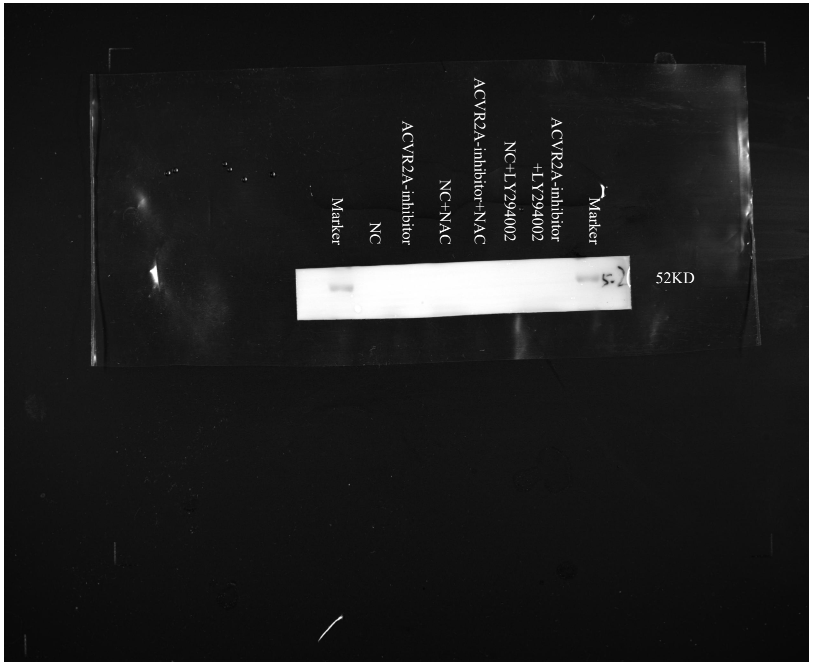

Fig.7A_CyclinA_shine

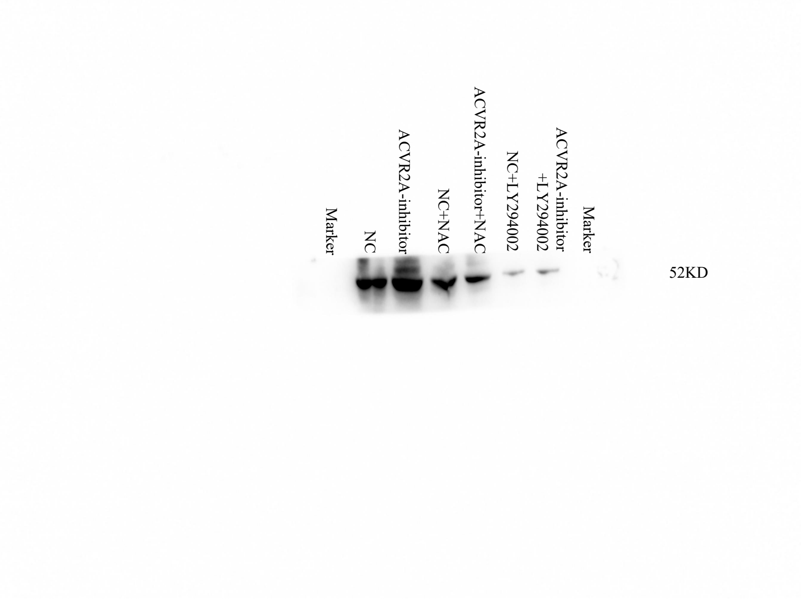

Fig.7A_CyclinA_merge

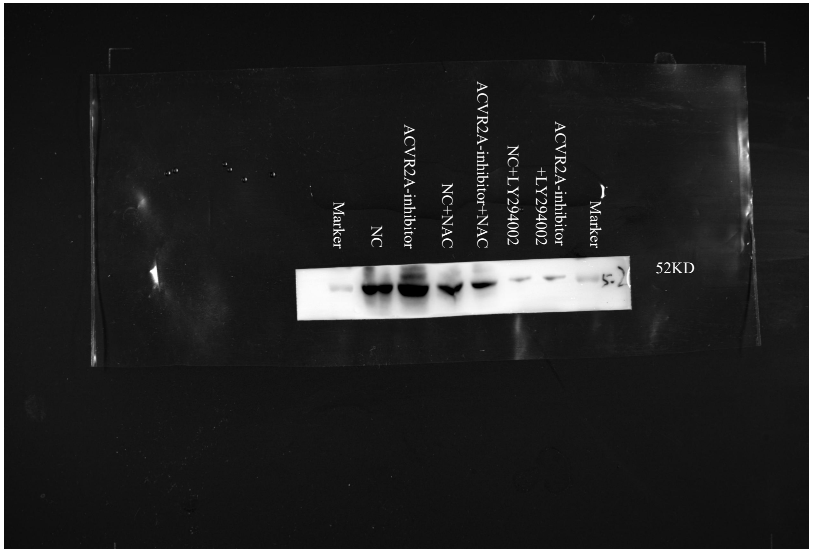

Fig.7A_CyclinD1_shine_raw

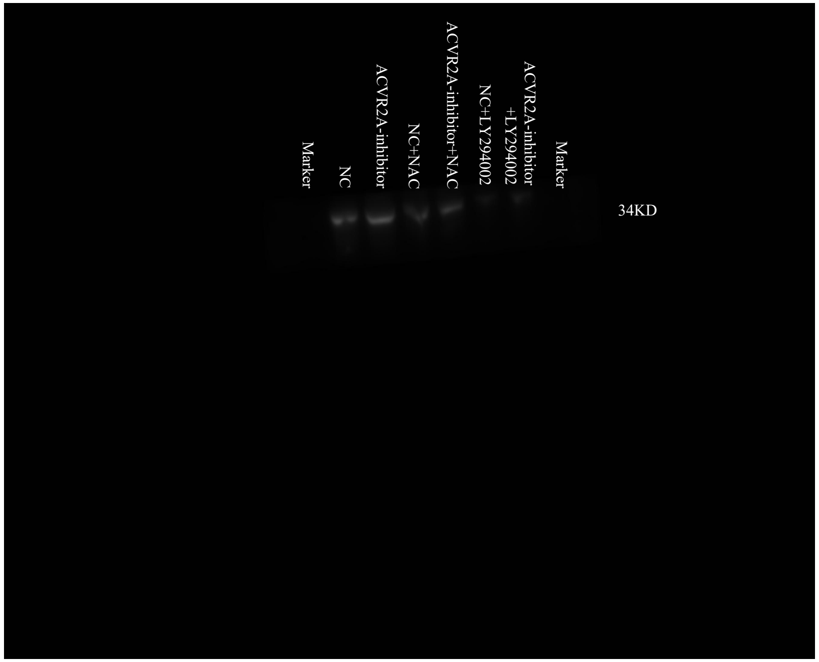

Fig.7A_CyclinD1_bright

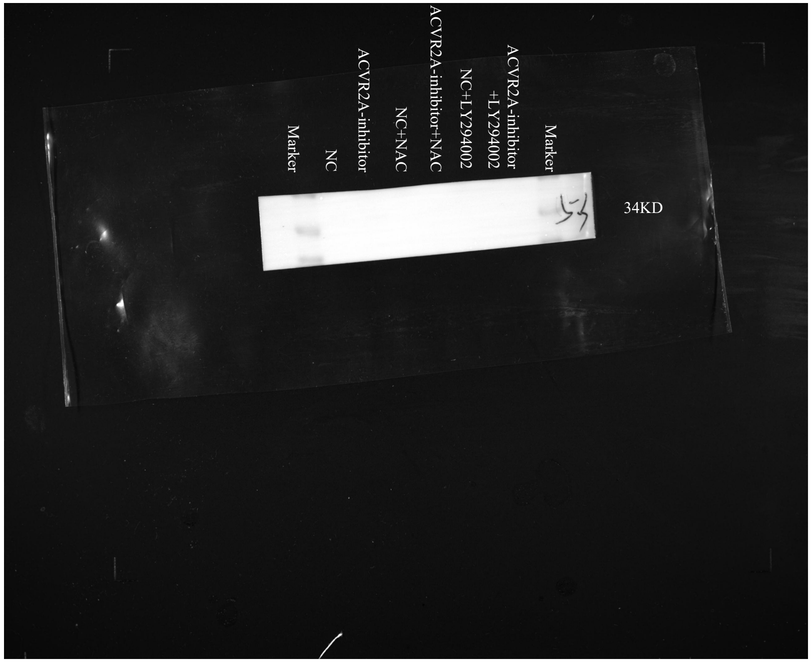

Fig.7A_CyclinD1_shine

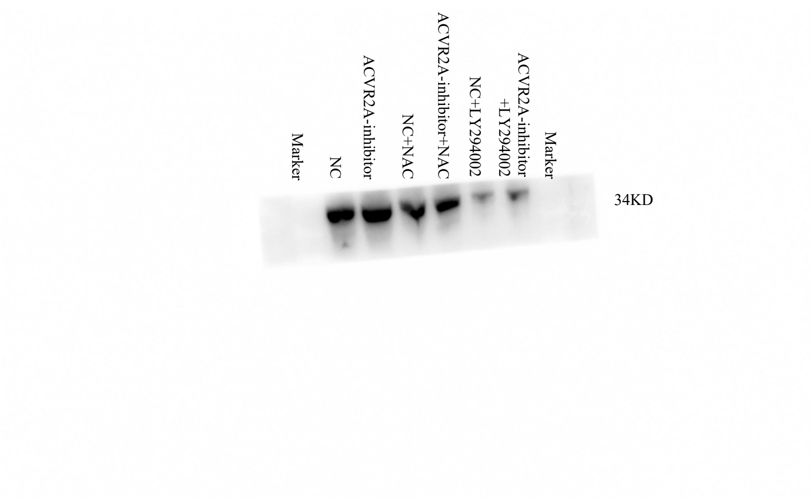

Fig.7A_CyclinD1_merge

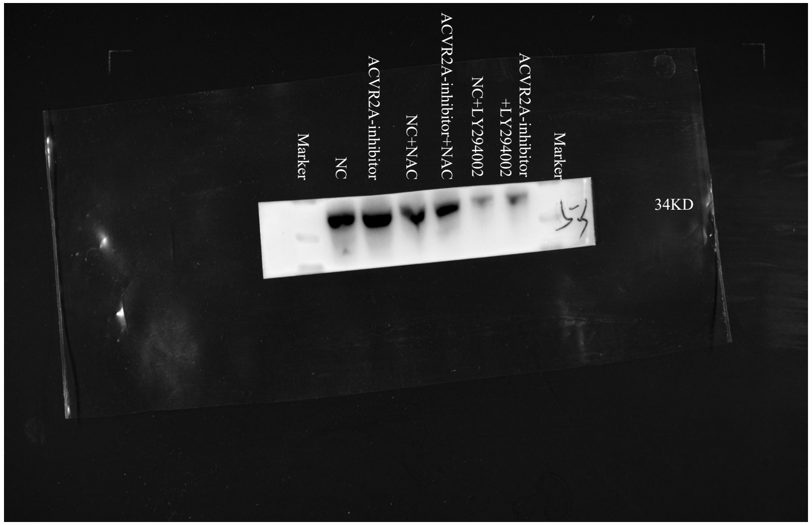

Fig.7A_HIF1α_shine_raw

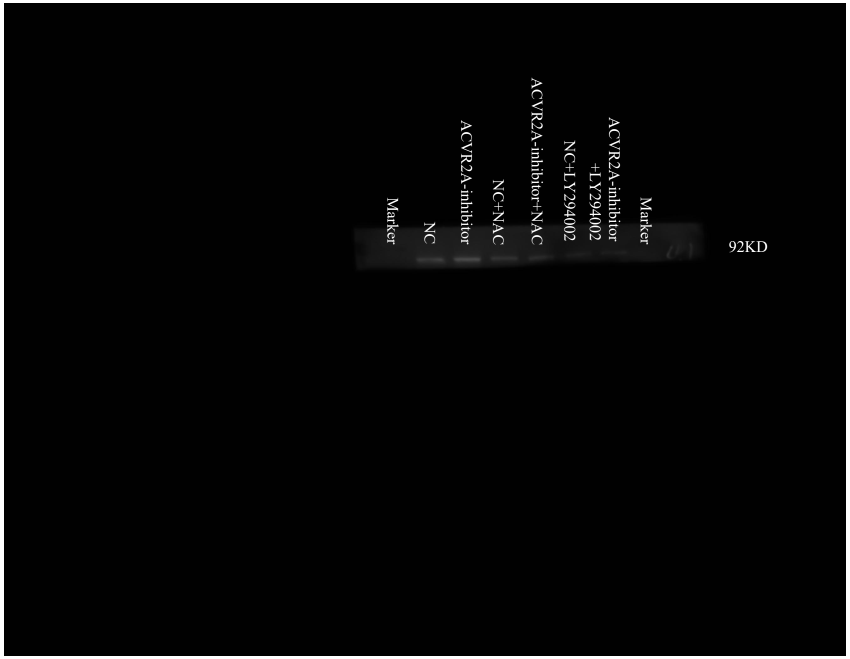

Fig.7A_HIF1α_bright

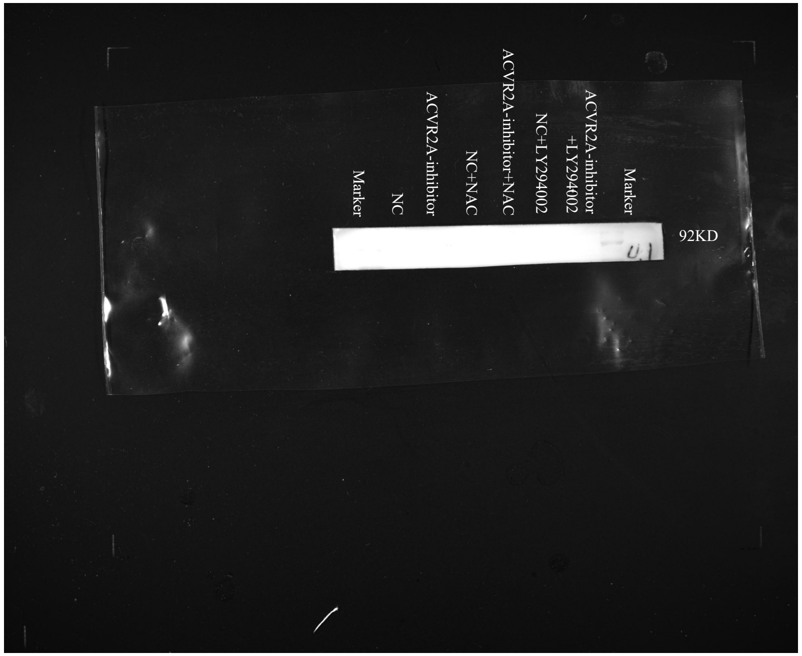

Fig.7A_HIF1α_shine

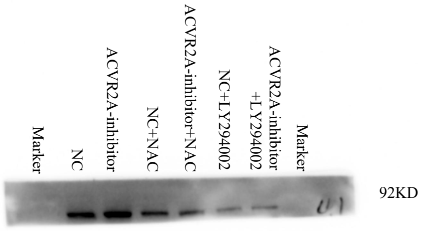

Fig.7A_HIF1α_merge

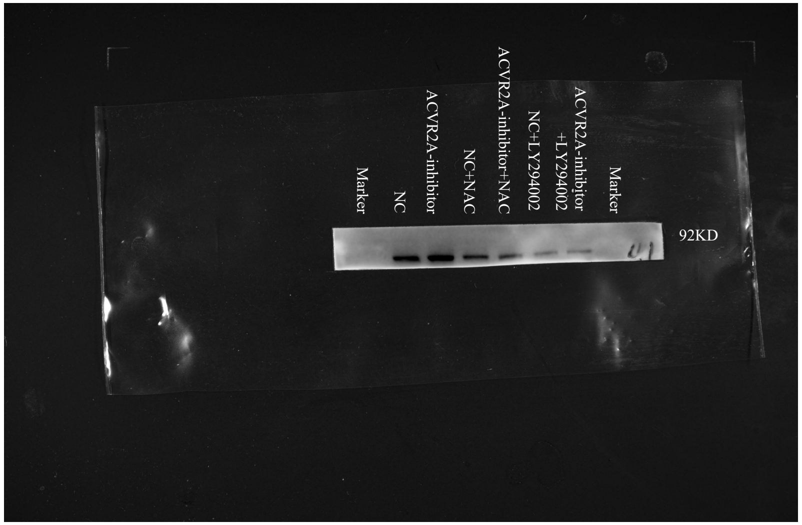

Fig.7A_GAPDH_shine_raw

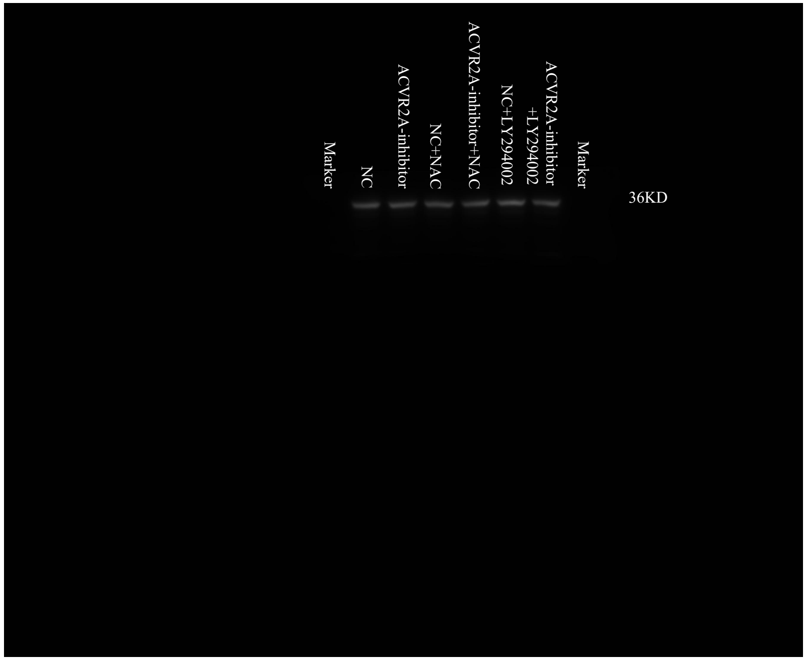

Fig.7A_GAPDH_bright

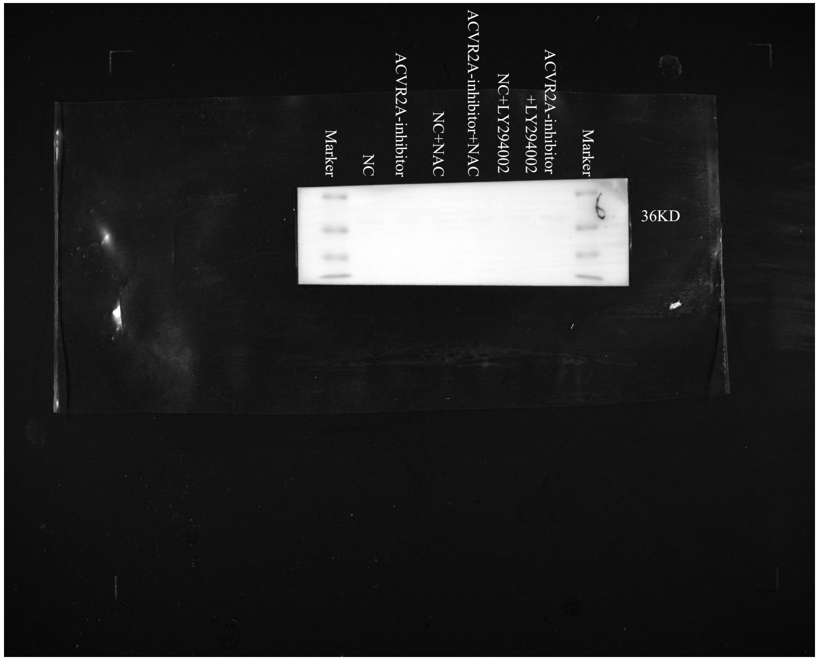

Fig.7A_GAPDH_shine

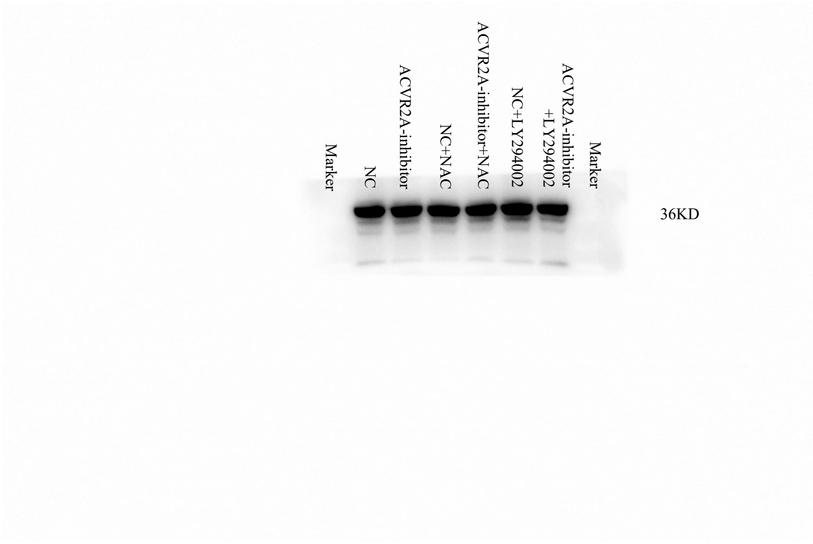

Fig.7A_GAPDH_merge

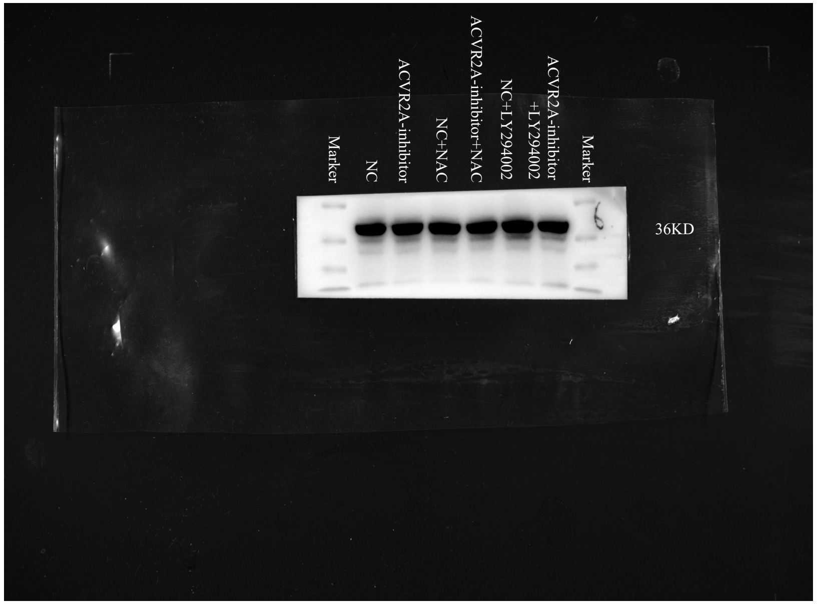

Supplement: Supplementary file 4 — Supplementary Information. [file 41598_2024_62753_MOESM4_ESM.pdf]
